# Interleukin-7 Receptor Alpha in Innate Lymphoid Cells: More Than a Marker

**DOI:** 10.3389/fimmu.2019.02897

**Published:** 2019-12-11

**Authors:** Abdalla Sheikh, Ninan Abraham

**Affiliations:** ^1^Department of Microbiology and Immunology, University of British Columbia, Vancouver, BC, Canada; ^2^Life Sciences Institute, University of British Columbia, Vancouver, BC, Canada; ^3^Department of Zoology, University of British Columbia, Vancouver, BC, Canada

**Keywords:** IL-7, TSLP, innate lymphoid cells, mucosal immunity, lymphopoiesis, inflammation

## Abstract

Innate lymphoid cells (ILCs) are a group of immune cells that are important for defense against pathogens, tissue repair, and lymphoid organogenesis. They share similar characteristics with various subsets of helper T cells but lack specific antigen receptors. Interleukin-7 (IL-7) and thymic stromal lymphopoietin (TSLP) are cytokines that engage the IL-7Rα and have major roles in dictating the fate of ILCs. Recent advances in the field have revealed transcriptional programs associated with ILC development and function. In this article, we will review recent studies of the role of IL-7 and TSLP in ILC development and function during infection and inflammation.

## Introduction

Innate lymphoid cells (ILCs) are a recently discovered subset of immune cells critical for the development of innate immunity against external pathogens, facilitating tissue repair, and mediating inflammation at multiple mucosal sites (1). It has become clear that these cells are major contributors despite being rare in proportion among immune cells. Although they lack antigen receptors, ILCs share multiple developmental circuitries with and are ancestral to the more abundant adaptive lymphoid cells. Both populations of lymphoid cells are known to develop from the same stem cell precursors in the bone marrow called common lymphoid precursors (CLPs) (2–5). In addition to sharing common progenitors with adaptive cells in the bone marrow, ILCs also require similar growth factors and cytokines to develop and function. The interleukin-7 receptor α (IL-7Rα or CD127) dependent cytokines, IL-7 and thymic stromal lymphopoietin (TSLP) are examples of such cytokines and they play an important role in determining the fate and function of ILCs (1, 6, 7). IL-7 is canonically important for the early development of B and T cells from bone marrow precursors and the thymic development of T cells (8, 9). Mature T cells also require IL-7 for survival, proliferation and multiple effector functions during infections and tumor infiltration (10–13). TSLP is mainly and constitutively produced by epithelial cells of the skin, gut and lungs, and shapes the response of dendritic cells and T cells against invading pathogens in a typical type 2 “weep and sweep” response that when misdirected, may also contribute to asthma and allergic inflammation (14–17).

Due to the heterogeneity in ILC populations and their multiple precursors, and our incomplete understanding of the biological factors (transcription factors, cytokines, disease states, etc.) that dictate ILC lineage commitment and function, we lack the knowledge to use ILC biology to develop new treatments or understand the precise role of ILCs in response to current therapeutics. For example, cytokines that govern adaptive lymphoid cells such as rhIL-7 are in multiple clinical trials for treatment of HIV, solid tumors, T cell reconstitution, and enhancing CAR-T cell therapy for B-cell lymphomas, yet the effects this may have on ILCs and other disease outcomes is understudied (18). In this review we will discuss relevant findings on the roles of IL-7 and TSLP in ILC development and function at various tissue sites as well as the mechanisms involved downstream of their signals. Where appropriate, we will also identify the significant gaps in the field and possible future directions.

## IL-7 and TSLP

**Figure 1:**
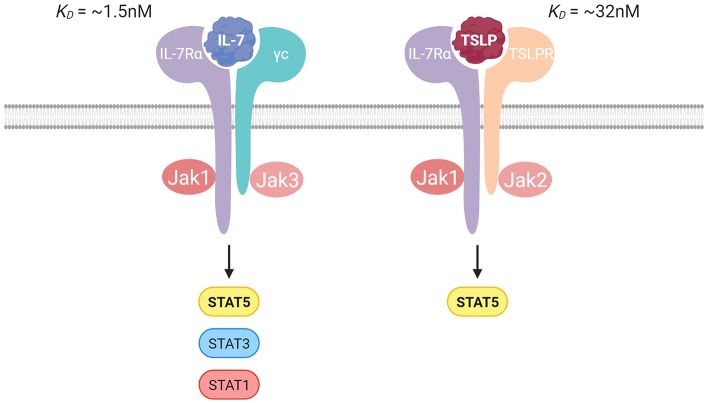
Illustration of the IL-7 and TSLP receptor complexes with the binding affinities. IL-7 signals through IL-7Rα paired with the γc receptor, and TSLP signals through IL-7Rα paired with the TSLP receptor. Jak/STAT signaling is crucial in transduction of signal for both cytokines.

IL-7Rα is found on multiple subsets of lymphoid cells during their developmental and mature states. Both IL-7 and TSLP use IL-7Rα to initiate the formation of a heterodimeric receptor. IL-7 is a common gamma chain (γc) cytokine and requires the heterodimerization of IL-7Rα with the γc receptor (CD132) for signaling (19), whereas TSLP signaling requires heterodimerization with the TSLP receptor (TSLPR) (20, 21) (Figure 1).

IL-7 is produced mainly by stromal cells in the bone marrow and thymus under steady state where it plays an indispensable role in the development of both pre- and pro-B cells in the bone marrow (22). The dynamic expression of IL-7Ra was shown to be critical for IL-7 responsiveness for specific stages of maturation in the thymus in shaping T cell development and survival (23–25). In addition to being an essential factor for B and T cell development, IL-7 can also influence effector T cells. For instance, exogenous IL-7 treatment enhances cytotoxic CD8 T cell anti-tumor activity and reverses T cell exhaustion caused by chronic LCMV infection, and thus, preventing liver pathology (10, 11).

While IL-7 is known to be produced mainly in the stromal and epithelial cells of the bone marrow and thymus respectively, the cells in the various other tissues that are capable of producing this cytokine are elusive (26). It is clear that the main producers are radio resistant cells of non-hematopoietic origin (27). IL-7 can be detected at low levels in the small intestine, lungs, liver, and skin where it can modulate T cell responses (28–32). In addition, through use of IL-7-eGFP mice, it became apparent that while stromal and epithelial cells contribute to IL-7 production, lymphatic endothelial cells are the key producers of IL-7 throughout the body including mucosal tissues such as the lung (33, 34). However, whether these cells are the primary source of IL-7 during inflammation, and if they play a role in ILC responses, remains a question.

What regulates the expression or availability of IL-7 in mucosal barrier tissues is not entirely clear. In the liver, LPS induced TLR stimulation leads to TRIF dependent expression of IL-7 in hepatic cells (28). Whether this mechanism is conserved in other tissues is not known. Interestingly however, while both ILCs and T cells express IL-7Rα, the expression of this receptor is significantly higher on ILCs due to an increased resistance to IL-7 mediated internalization (27). As such, ILCs are key regulators of the availability of IL-7 in lymphoid tissues acting as a cytokine sink and limiting IL-7 availability to other cells such as T cells which depend on IL-7 for homeostatic proliferation (27). Considering T cells heavily outnumber ILCs, further investigation is necessary to determine the extent to which ILCs limit the availability of IL-7 in peripheral tissues and how that affects other immune cells during homeostasis and inflammation.

TSLP was originally identified in a conditioned murine thymic stromal cell line supernatant and reported to play a role in the *in vitro* development of B cells (35–37). However, loss of function *in vivo* studies have demonstrated minimal or secondary role for TSLP in lymphoid development (22, 38, 39). IL-7 and TSLP are often compared with each other owing to their shared dependency on IL-7Rα and ability to activate STAT5, albeit through different JAK proteins (40). However, despite its discovery from thymic cells and its nomenclature, TSLP is mainly produced in a constitutive manner by epithelial cells, notably by keratinocytes and that of mucosal organs such as the intestine and lungs (14, 15, 41, 42). There is strong evidence suggesting its importance in maintaining barrier integrity in these locations upon infection and inflammation through tissue remodeling and by conditioning dendritic cells (DCs) toward a tolerogenic phenotype. This supports the development of regulatory T cells and polarization of activated helper T cells to exhibit type 2 (Th2) characteristics (14–17). Altogether, these findings suggest roles for TSLP that are not only segregated spatially and possibly temporally from that of IL-7 but also serve a unique function in developing immune responses.

## ILC Subsets and Their Origin

Terminally differentiated ILCs closely resemble helper T cells, such that a version of a helper ILC mirrors each of Th1, 2, 17 helper T cells in terms of key transcription factor dependency, cytokine output, and resulting pathologies (43, 44). In fact, most of what we have learned about the function and development of ILCs since their discovery has been aided by our knowledge of T cells.

ILCs have been documented and categorized into three general helper ILC groups. Group 1 ILCs (ILC1s) are found in various tissues including the small intestine and liver, are dependent on T-bet and produce the Th1 cytokines IFN-γ and TNF- α (4, 45). Natural killer (NK) cells are similar to ILC1s but are not considered part of the helper ILC subset and generally do not express the IL-7Rα (45). However, some tissue NK cells express a range of IL-7Rα levels, like in the thymus, colon, and small intestine lamina propria (siLP) (46, 47). Group 2 ILCs (ILC2s) express GATA3 prominently, similar to Th2 cells, and likewise, produce IL-5, IL-13, and in some conditions IL-9 when activated by the alarmins TSLP, IL-33, and IL-25 (6, 48–50). They are also the major ILC subset found in the lungs and play an important role in airway immunity (51, 52). Group 3 ILCs (ILC3s) are RORγt-dependent, like Th17 cells, and consist of a major subset that produces IL-17 and IL-22 in response to IL-23 and are critical for intestinal immunity against pathogens (1, 53). Another subset of ILCs are the Lymphoid Tissue inducer cells (LTi), which are also RORγt-dependent group 3 ILCs, and are considered important for the development of secondary lymphoid organs (54).

In mice, the fetal liver serves as the earliest known source of ILCs where IL-7 is known to be produced and support the development of other lymphoid cells (5, 55). The various precursors at different stages of ILC development are primarily studied using mouse models and are classified based on their surface markers and the transcription factors that lead to their lineage restriction. Common lymphoid progenitors (CLPs) are descendants of lymphoid-primed multipotent progenitors (LMPPs) which largely do not express IL-7Rα and are the source of all lymphoid cells (56). CLPs can develop into all lymphoid cell progenitor subsets including α-lymphoid progenitors (αLP), early innate lymphoid progenitors (EILPs), and the common helper innate lymphoid precursors (CHILPs) (57, 58). αLP and EILPs can develop into all of the known ILC subsets including ILCs 1, 2 and 3 and conventional NK cells (57, 58). These cells are also known as global innate lymphoid progenitors (GILPs). The more restricted precursors, Id2^+^ common helper innate lymphoid precursor (CHILPs), can generate all helper ILC subsets (ILC1, 2, 3 and LTi) but not NK cells (4). LTi cells arise from PLZF^−^ precursors while the rest of the helper ILCs 1, 2, and 3 arise from PLZF^+^ innate lymphoid precursors (5). However, it is important to note that a hierarchical model of development is prone to revision based on new studies. The developmental stages and potentials of the various ILC precursors are more nuanced and complex, and can change depending on the organism, age, sex, inflammation, and the tissues examined (55).

When helper ILCs were discovered as a new subset of immune cells and reported to play important roles in immunity, they were described as IL-7Rα^+^ cells. Aside from being an important defining marker for a major subset of ILCs, IL-7Rα is important in mediating IL-7 and TSLP signaling in these cells to promote their development and function.

## IL-7Rα in the Development of ILCs

### IL-7 and ILC Development

**Figure 2:**
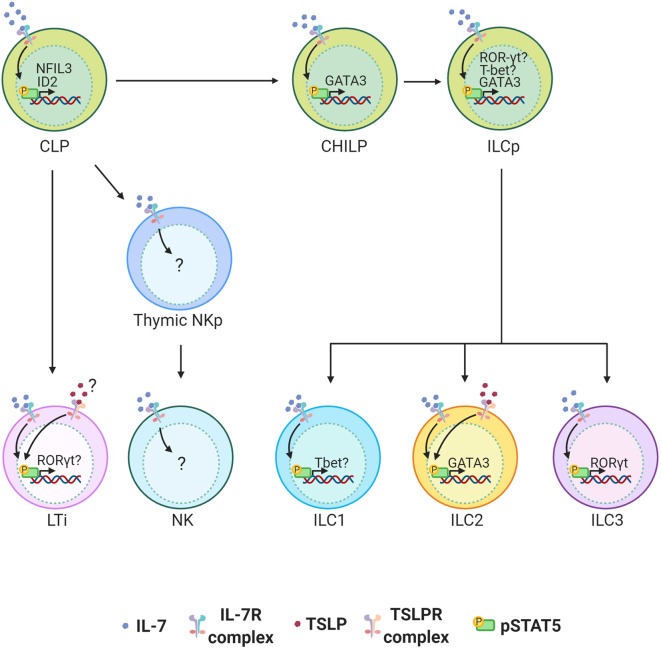
ILC development in the bone marrow (BM) is highly dependent on IL-7 signaling. IL-7 engagement with the IL-7 Receptor complex activates STAT5. TSLPR expression on BM ILC precursors is not clear and its functional role in the development of ILCs is minimal. STAT5 activation by IL-7 induces NFIL3 in common lymphoid progenitors (CLPs) in the BM of adult mice and this is required for the expression of ID2 and generation of all helper ILCs. STAT5 is also important for the induction of GATA3 which can play an important role in the development of all ILCs at any stage between common helper innate lymphoid precursors (CHILP) and innate lymphoid precursors (ILCPs) to group 2 innate lymphoid cells (ILC2s). In ILCs STAT5 also induces RORγt and Tbet. The extent to which this plays role in terminal differentiation of ILCP to ILC 1, 2 and 3 is not clear. While IL-7 is critical for the development of LTi cells and thus lymph nodes, TSLP can partially compensate for the loss of IL-7.

IL-7 is indispensable for the development of all helper ILCs. The role of IL-7 in ILC development was initially discovered in LTi cells. It is now well-established that IL-7 is important for LTi cell development and therefore the architecture of secondary lymphoid organs (59–61) [reviewed in (62).] The more recent discovery of the helper-ILC groups extended the importance of IL-7 to the development of other ILCs. It was first reported that “natural helper” cells (now called ILC2s) associated with the adipose tissue depend on IL-7 to survive and maintain their numbers in the tissue (6). Despite these findings, we have been unaware of how IL-7 instructs the development of ILCs. The recent unveiling of the heterogeneity in ILC precursors and their transcription factor dependency has now led to a better understanding of the factors that mediate the development of ILCs. Nuclear factor IL-3 (NFIL3) is a transcription factor required for NK cell development, and it was more recently found to be critical for the development of all other ILCs (4, 58, 63–66). The expression of NFIL3 in CLPs requires IL-7, which directs STAT5 activation and binding of pSTAT5 to the NFIL3 promoter (63). NFIL3 expression is specifically required for Id2 expression and generation of CHILPs, and hence the development of all helper-ILCs (63). While it is better established that IL-7 controls pan helper-ILC development through control of a common precursor from CLPs to CHILPs, it is less clear how IL-7 controls development of more committed PLZF^+^ ILC precursors (ILCPs) from CHILPs. It is known that expression of GATA3 at the CHILP stage is required for the development of ILCPs that give rise to the majority of the ILC lineage (67). Since GATA3 is downstream of STAT5, it is possible that IL-7 signaling is important if not indispensable in the development of ILCPs and their ILC2 progenies through related pathways (68, 69). In addition to inducing GATA3 in ILC2s, a recent studies have found STAT5 to be a major regulator of ILC homeostasis by regulating multiple networks ranging from survival factors such as Bcl-2 and transcription factors such as T-bet, RORγt, and Sall3 which each play a role in the function and differentiation of various ILC subsets (70, 71) (Figure 2).

In addition to the BM, IL-7 is produced in the fetal liver where earliest progenitors of ILCs can be found (72). Moreover, IL-7 is known to orchestrate the development of lymphoid cells in this tissue (73). IL-7 is also abundant in the thymus where T cells develop from early thymic progenitors (ETPs). Increasing evidence suggests thymic development of ILCs, however, it is unclear to what degree the thymus serves as a source of ILCs and whether ILCs and T cells share the same thymic progenitors. Early studies describe a mouse thymic NK cell subset that expressed the IL-7Rα depends on IL-7 for their development and has the capability to seed peripheral tissues (47). These murine thymic NK cells also shared similar characteristics to human CD56^+^ NK cells (47). More recent studies have shown E-proteins E2A and HEB to be specifiers of T cell commitment in thymic precursors and their deletion leads to a skew toward ILC2 development, suggesting that thymocytes explore multiple fates before commitment (74, 75). Furthermore, IL-7Rα expression is inversely correlated with the expression of E-proteins in early ILC progenitors in the BM but correlates directly with that of Id2. This suggests an increased dependence on IL-7 for ILC development. To better understand the role of IL-7 in thymic ILC development, it is important that we elucidate its downstream signaling in various ILC precursors in the BM and thymus.

IL-7 has recently been found at the center of ILC differentiation. For instance, the quantity of IL-7 that is available combined with the strength and duration of Notch signaling can dictate the fate of mouse fetal liver derived CLPs (76). Whereas, high IL-7 and medium Notch signaling favors ILC2 and ILC1/NK cell differentiation, low IL-7 and high Notch signaling favors T cell differentiation in mice (76). The results are however different when human hematopoietic progenitor cells are treated in a similar manner, where IL-7, and Notch signaling induce ILC3 differentiation while suppressing IL-15 induced NK cell differentiation (77). These findings reveal contrasting roles for IL-7 in ILC differentiation which can be explained by differences in mouse and human hematopoiesis and progenitor sources. Further studies are needed to identify other contributing factors that create divergent roles for IL-7.

While IL-7 is clearly crucial to the development of ILCs, other cytokines can partially compensate for IL-7 or drive IL-7-independent maintenance of ILCs. For instance, IL-15 (a γc cytokine) can partially complement IL-7's defects in ILCs in IL-7Rα^−/−^ mice (46). This is based on a study that found residual ILCs in IL-7Rα^−/−^ mice express the IL-15Rβ and respond variably to IL-15 *in vitro* through increased survival depending on the type of ILCs (46). Further assessment of IL-15^−/−^, IL-7Rα^−/−^, and IL-7Rα^−/−^ IL-15^−/−^ mice revealed IL-7-independent sustenance of ILCs by IL-15 that varied in degrees depending on the type of ILCs and the tissue examined. Most notably, NK cells were normal in numbers in IL-7Rα^−/−^ mice but greatly reduced in IL-15^−/−^ mice as expected (46). However, IL-7Rα^−/−^ IL-15^−/−^ mice had greater loss of NK cells compared to IL-15^−/−^ mice, more so in the colon than the siLP. Consistent with this is the higher expression of IL-7Rα observed by colon NK cells compared to siLP NK cells. This suggests a supportive role for IL-7 in NK cell development in a tissue specific manner. ILC1s were only marginally reduced in IL-7Rα^−/−^ and IL-15^−/−^ mice compared to WT mice, while IL-7Rα^−/−^ IL-15^−/−^ mice experienced a multi-fold reduction in ILC1s in the colon and the siLP, suggesting a synergistic contribution by both cytokines in development/maintenance of ILC1s (46). While IL-15^−/−^ mice have normal number of NKp46^+^ and CCR6^+^ ILC3s in the siLP and colon, loss of both IL-7 and IL-15 signaling results in even greater loss of these cells compared to IL-7Rα^−/−^ mice. Similarly, IL-7Rα^−/−^ IL-15^−/−^ mice had greater reduction in number of ILC2s in the siLP and colon than IL-7Rα^−/−^ mice. However, IL-7Rα^−/−^ and IL-7Rα^−/−^ IL-15^−/−^ mice have equal numbers of ILC2Ps suggesting a supportive role for IL-15 in survival of ILC2s in the periphery (46). The subset of ILC2s most affected in IL-7Rα^−/−^ mice were ST2^+^ KLRG1^+^ ILC2s while ST2^−^ ILC2s were unaffected. The functional importance of these residual ILC2s has yet to be determined. Since they lack ST2 (IL-33 receptor) and are IL-7Rα deficient, they are non-responsive to IL-33, IL-7, and TSLP—the most potent activators of ILC2s—what are the cytokines that activate these cells? Common gamma chain cytokines including IL-7 and IL-15 use the γc receptor which relies on Jak3 to transmit signals. Loss or mutation of this receptor leads to loss of multiple ILC subsets in mice and inhibition of Jak3 using tofacitinib abrogates human ILC1 and 3 proliferation and development *in vitro* (78). Investigating downstream signaling factors can help identify overlapping pathways that are necessary for the development of ILCs.

FLT3 ligand (FLT3L) can also compensate for IL-7 in ILC development (79). IL-7^−/−^ mice present with normal numbers of NK cells in the small intestine but have reduced ILC2s and ILC3s. Loss of FLT3L depletes all ILCs including NK cells suggesting a role for FLT3 that is earlier than that of IL-7 in ILC development (79). Treatment with rFLT3L for 10 days can restore all ILC populations in of IL-7^−/−^ mice except for ILC2s which suggests either a greater dependence on IL-7 by ILC2s or that FLT3L mediated rescue of ILCs occurs at later stages of ILC development, perhaps after commitment to specific groups.

### TSLP and ILC Development

The influence of TSLP in the development of ILCs is minimal, as previous reports using TSLPR^−/−^ mice have shown normal numbers of ILC2s in the lungs (80). This is not surprising since its effect in lymphopoiesis is insubstantial as well. Although loss of TSLP signaling has little effect on lymphopoiesis, addition of TSLP to *in vitro* cultures can enhance mouse and human B cell as well as mouse T cell expansion from hematopoietic progenitors sourced from fetal liver (37, 81–83). It is however, unclear if ILC progenitors express the TSLPR and if significant levels of TSLP are produced in the fetal liver and bone marrow.

Both IL-7 and TSLP use the IL-7Rα but the increased importance that IL-7 has compared to TSLP in lymphoid development may stem from the increased binding affinity of IL-7 to its receptors, or more likely due to the established importance of γc/Jak3 in ILC development. Understanding how well TSLPR and/or Jak2 facilitate ILC development will be important to make a definitive statement.

Most studies of TSLP are in the context of ILC2s since no other ILCs have been reported to express the receptor for TSLP. Nonetheless, ILC research is in its preliminary stages and identification of the transcriptional dependencies of the various ILC subsets is in progress. It is possible that TSLP can mediate aspects of ILC development through unidentified pathways that are possibly masked by our current method of grouping ILCs. Supporting this hypothesis, over expression of TSLP has been shown to support the development of LTis in a compensatory manner in IL-7-deficient mice (61) (Figure 2). It is unknown whether these effects by TSLP are direct or indirect. Since the manipulation of TSLP and/or IL-7 signaling is integral for drugs that treat several conditions including allergies, cancers and infectious diseases, it is important that we have a better understanding of the interplay between the two cytokines in ILC development to design more efficient drug treatments (18, 84).

## IL-7Rα in ILC Homeostasis and Function

### IL-7 and ILC Function

While studies on IL-7's developmental roles in ILCs are substantial, research in its effector functions are relatively modest. Indeed, IL-7 was considered an important factor for the development of T cells long before its role in effector functions was examined. IL-7 is mainly produced in primary lymphoid tissues such as the bone marrow and thymic stromal cells where immune cell development occurs (26, 85). However, this cytokine is also produced in secondary lymphoid organs and can be induced in skin, lung, intestinal epithelial cells, and liver as shown by fluorescence microscopy of IL-7 reporter mice and ELISA (28, 29, 32, 85, 86). The extent of our knowledge on IL-7 and ILC function is based on a series of *in vitro* experiments.

**Figure 3:**
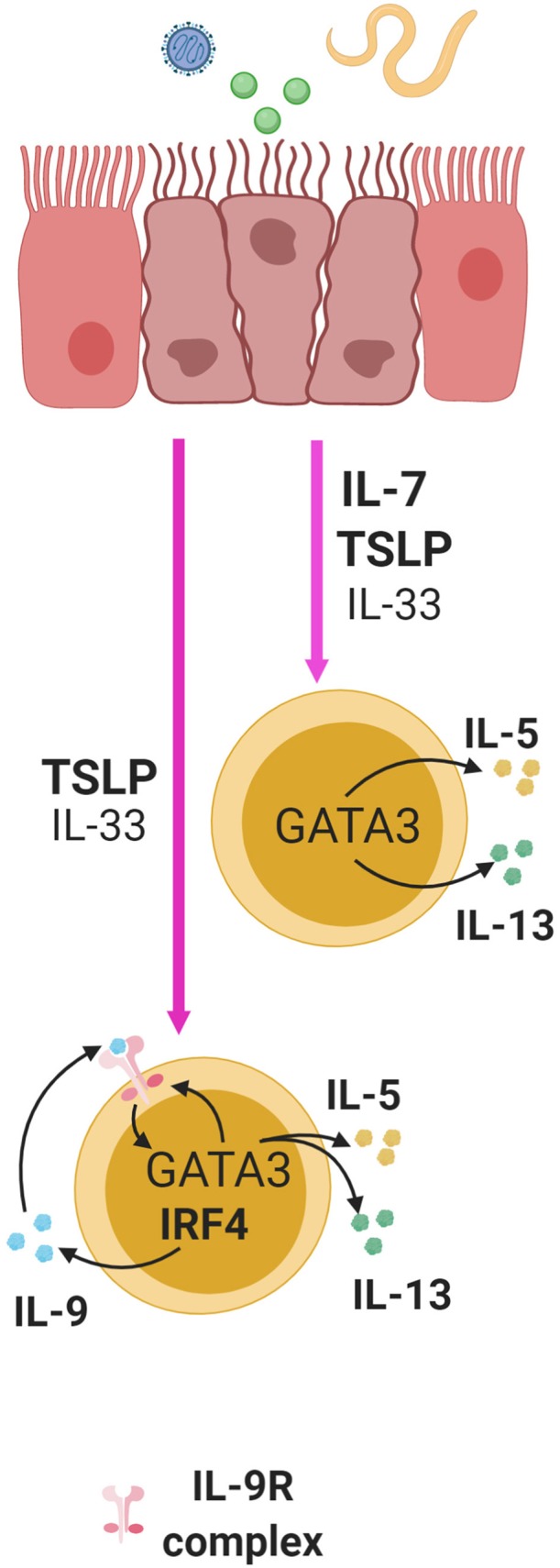
ILC2s in the lung, small intestine, and skin are activated by alarmins following exposure to virus, helminthes, or allergens and this induces cytokine production in ILC2s. IL-7 and TSLP significantly enhance the effect of IL-33 in multiple mucosal sites. TSLP and IL-33 synergistically induce the expression of IL-9 in an IRF4 dependent manner in ILC2s. Through autocrine signaling, this can enhance the production of IL-5 and IL-13 and IL-9R in a GATA3 dependent manner, thus creating a positive feedback loop to further enhance cytokine production.

IL-7 alone or with IL-33 can stimulate the production of Th2 cytokines, IL-5 and IL-13, from murine ILC2s (6, 48, 50, 87) (Figure 3). These Th2 cytokines are important factors produced by ILC2s that promote helminth expulsion, antiviral effects, and tissue repair. A recent study showed that mice lacking T-bet had increased number of ILC2s and production of IL-5 and IL-13 leading to enhanced worm clearance during a *Trichinella spiralis* infection (88). This activity in ILC2s correlated with higher expression of IL-7Rα leading to increased activation of STAT5 (88). This suggests that T-bet is a regulator of IL-7Rα expression, and that IL-7 may enhance ILC2 function (88). This necessitates further *in vivo* examination of the role of IL-7 in ILC2 function.

**Figure 4:**
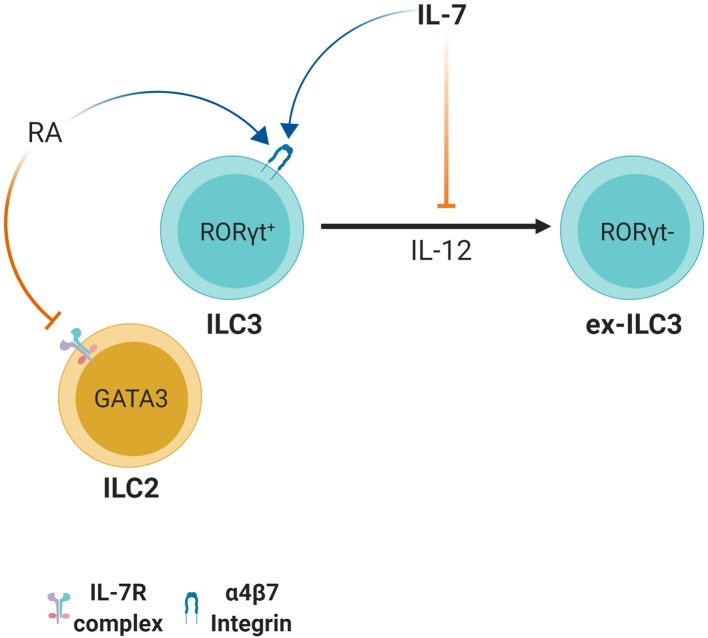
Retinoic acid and IL-7 induce the expression of the gut homing receptor integrin α4β7. However, in ILC2s, retinoic acid causes downregulation of IL-7Rα. ILC3s appear to have plasticity since stimulation with IL-12 leads to conversion to ILC1-like ex-ILC3s. Aided by microbial stimulation, IL-7 induces or stabilizes RORγt expression and maintains the IL-22 production status of ILC3s thus preventing conversion to ILC1-like ex-ILC3s.

RORγt^+^ ILC3 derived IL-22 plays an important role in defense and regulation of pathogenic and non-pathogenic bacteria in the intestine by maintaining barrier integrity and inducing anti-microbial peptide expression by epithelial cells such as RegIIIγ and RegIIIβ (89–91). RORγt^+^ ILC3s can however lose these abilities and differentiate into RORγt^−^ cells through stimulation with IL-12 and IL-15 (92). This leads to their conversion from IL-22 producing ILC3s to IFN-γ producing ILC1-like cells. Together with the microbiota, IL-7 is able to counteract this transition by stabilizing RORγt expression (92) (Figure 4). IL-23 is the key cytokine responsible for RORγt-mediated IL-22 production by ILC3s (93). This finding suggests that IL-7 may in part be important for maintaining the IL-22 production status in ILC3s, which is pivotal for host defense and barrier integrity during bacterial infection. Indeed, IL-7 stimulation can induce RORγt expression and play a supportive role in IL-23 mediated IL-22 production in ILC3s (94). Furthermore, *in vitro* co-culture of IL-7 producing mesenchymal stromal cells (MSCs) with ILC3s led to IL-22 production and enhanced IL-2 induced proliferation of ILC3s (95). This was due to IL-7 derived from MSCs as measured by ELISA (95).

IL-7, together with retinoic acid (RA), is also important for homing of ILCs to gut-associated tissues by upregulating ILC3-intrinsic expression of α4β7 and selectin ligands, an effect mediated by IL-7 in T cell homing as well (96, 97) (Figure 4). Interestingly, IL-7 is also required for survival of ILC3s in the LN post-development, which in turn is important for homing naïve T cells to lymphoid tissues (98). It should be noted however, that while the RA/IL-7 axis has a positive effect on ILC3s, treatment with RA reduces IL-7Rα expression in ILC2s and ILC2Ps and this leads to reduced numbers of ILC2s possibly through reduced survival, homing or development (99) (Figure 4). Taken together, IL-7 plays diverse roles in dictating ILC function but new approaches are necessary to clarify them and clearly distinguish them from IL-7's developmental roles.

### TSLP in ILC2 Function

TSLP, along with IL-33 and IL-25, are produced by mucosal epithelial cells, and as alarmins, these cytokines are important activators of immune responses. Stimulation of lung epithelial cells with virus, allergens or helminths can lead to enhanced production of TSLP, IL-33 and IL-25, and stimulation of lung ILC2s with TSLP alone or in combination with IL-33 can induce IL-4, IL-5 and IL-13 secretion (48–50, 80). Interestingly, while IL-33 alone is able to induce such secretion *in vitro*, adding small doses of TSLP to the stimulation cocktail is sufficient to significantly enhance the secretory program, indicating its potency (50) (Figure 3). Furthermore, TSLP is able to mediate skin inflammation through ILC2s in mice independent of IL-33 and IL-25 (100). In summary, TSLP can greatly enhance ILC2 responses with or without help from IL-33 and IL-25.

#### The TSLP-GATA3 Axis in ILC2 Function and Homeostasis

TSLP can activate multiple signaling pathways in T cells and ILC2s alike. In T cells, TSLP as well as IL-7, are able to activate STAT5 signaling which activates pro-survival signals mediated by Bcl-2 (40). Similarly, ILC2s respond to TSLP by activating STAT5, and this triggers IL-13 production (68). The transcription factor GATA3 is indispensable for the development of all ILCs, an important identifying marker for ILC2s, and a direct target of STAT5 (67, 68). In addition, TSLP can induce GATA3 in ILC2s which in turn mediates IL-4, IL-5 and IL-13 production *in vitro* in these cells, and silencing GATA3 alone greatly reduces this TSLP response (68). This signifies the importance of GATA3 for TSLP-mediated ILC2 function. Interestingly, GATA3 induction can lead to enhanced expression of TSLPR and IL-33 receptor (ST2) which suggests that TSLP signaling through GATA3 can enhance responsiveness to IL-33 and TSLP (68). This model is consistent with studies that have suggested TSLP and IL-33 having synergistic effects on ILC2s (50). Moreover, GATA3 can directly bind to exon 2 of the gene encoding IL-7Rα and induce its expression thus enhancing IL-7 and TSLP signaling (101). Altogether, assuming linearity, this suggests a positive feedback loop whereby TSLP and GATA3 signaling rely on each other to amplify early innate responses by relatively rare cells in an environment with limited cytokine availability.

#### TSLP in Support of IL-9 Programming of ILC2 Function

ILC2s can produce IL-9 in response to TSLP in a manner dependent on the transcription factor interferon regulatory factor 4 (IRF4) (50). IL-9 derived from ILC2s can also serve to enhance early ILC2 responses by signaling in an autocrine fashion to increase IL-5 and IL-13 production during helminth infection in mice with *Nippostrongylus brasiliensis* (Figure 3). This subsequently promotes the expression of genes important for mucus production and tissue repair in lung epithelial cells (50, 102). Additionally, IL-9 receptor expression has been found to be positively regulated by GATA3 through RNA-sequencing transcriptomic analysis of ILC2s (67). Since TSLP can induce GATA3 expression in ILC2s, this suggests that TSLP may shape ILC2 responses through control of IL-9 receptor and ligand expression (68) (Figure 3). This further demonstrates that TSLP can act through multiple pathways in regulating ILC2 function.

### The Dark Side: TSLP Duality

Notwithstanding its importance at barrier sites, TSLP is well-established as an inducer of Th2 cytokine driven allergic inflammation such as atopic dermatitis, eosinophilic esophagitis, asthma and allergic rhinitis, all mediated by a variety of cells including eosinophils, basophils, and ILC2s (16, 48, 100, 103, 104). TSLP supports the production of IL-5 and IL-13 by lung ILC2s, which contributes to eosinophilia and elevated mucus production in papain and chitin models of allergy induction (48, 50). Corticosteroids are a common treatment for asthma, but TSLP can enhance the survival and proliferation of IL-13^+^ ILC2s through STAT5 activation, thus limiting therapeutic effectiveness (105–107). Similarly, respiratory syncytial virus (RSV) infection in mice induces airway hyper responsiveness (AHR) and airway obstruction, characterized by enhanced ILC2 proliferation and production of IL-13, and this effect is significantly reduced in TSLPR-deficient mice (49). Interestingly, TSLPR protein expression can be upregulated in ILC2s early during RSV infection. Proliferating ILC2s that produce IL-13 (but not IL-5) had higher expression of TSLPR mRNA and protein. (49). This differential expression by subpopulations of ILC2s may explain how TSLP can have both beneficial and detrimental effects on ILC2s. Nonetheless, comparative analysis of AHR, allergy, and infection models is necessary for a definitive statement.

There are other circumstances that can lead to duality in TSLP's effect on ILC2s or other TSLPR expressing cells. Relevant clinical studies have implicated the over-production of TSLP, either due to mutation(s) or constant exposure to allergen(s), as the main culprit in TSLP-mediated allergic inflammation (108, 109). Another model suggests that TSLP may have diverse roles due to the presence of two transcript variants for TSLP producing a long and short isoforms (110, 111). The short isoform of TSLP is expressed constitutively during homeostasis and is important for anti-inflammatory, barrier integrity and anti-microbial responses, while the long isoform is expressed during inflammation and supports inflammatory cytokine production (110, 111). Lastly, it is possible that the combined effects of other cytokines in the environment with TSLP can influence the outcomes, and these varying compositions may define a certain threshold. One or more of these scenarios may occur simultaneously making multi-faceted approaches a preferred route of treatment for allergic inflammations.

The negative effects of TSLP have been noted in ILC3s as well. In a report, loss of IKKα in murine intestinal epithelial cells (IECs) led to an overproduction of TSLP during *Citrobacter rodentium* infection (112). This resulted in reduced IL-22 production by ILC3s, impaired bacteria clearance and increased mortality. *In vivo* blockade of TSLP was sufficient to restore anti-bacterial immunity. The inhibitory effect of TSLP on ILC3 function was confirmed in *in vitro* experiments. Addition of TSLP impaired the ability of IL-23 to stimulate IL-22 production from ILC3s in bulk splenocyte cultures, however this phenomenon was not seen with sort purified ILCs, suggesting that TSLP acted indirectly. This finding is unprecedented and warrants further investigation to clearly map the connection between TSLP and ILC3s, and provide more insight into their implications in mucosal and barrier health.

## Concluding Remarks

IL-7Rα is a cytokine receptor whose expression is tightly regulated throughout the development and life of lymphoid cells. IL-7 and TSLP signal through IL-7Rα and play multiple roles in determining the fate of ILCs and T cells. Since their discovery, research on ILCs have led to great insights in mucosal immunology and lymphoid development. Their resemblance to adaptive lymphoid cells has enabled us to study their biology more efficiently. Despite the current progress, we have yet to fill significant gaps of knowledge in ILC development and function. It is still unclear when commitment to the ILC fate occurs during hematopoiesis and how IL-7 controls this program. The development of ILCs *in vivo* was found to rely on key transcription factors such as NFIL3 whose activation is dependent on IL-7 signaling. However, without a complete picture of the source of ILCs, it is hard to pinpoint dependencies on any given single cytokine. In addition, recent studies have allowed us to better appreciate the complexity of hematopoiesis, and in doing so, the differences between murine and human lymphopoiesis. Although studies with mouse models have provided great insight in lymphopoiesis, we should be cautious in our interpretations. Recent advances in understanding ILC development can be credited to transcriptomic studies and single cell resolution analysis that have provided a complex view of ILC heterogeneity. Further studies utilizing similar methods can be conducted to identify ILC precursors in lymphoid and non-lymphoid tissues and examine the factors that regulate their development. Multiple studies that have shown a role for IL-7 and TSLP in ILC function have used *in vitro* treatment with cytokines. While these studies have provided great insights on how these cytokines can influence ILCs, it is important to validate and extend these findings through various transgenic animal models to reveal any physiologically relevant and indispensable roles of IL-7 and TSLP in ILC biology.

## Author Contributions

AS reviewed the literature and wrote the manuscript, edited it and generated the figures. NA reviewed the drafts, provided critical input and edited the text and figures.

### Conflict of Interest

The authors declare that the research was conducted in the absence of any commercial or financial relationships that could be construed as a potential conflict of interest.
